# Lipoprotein(a), Cardiovascular Events and Sex Differences: A Single Cardiological Unit Experience

**DOI:** 10.3390/jcm12030764

**Published:** 2023-01-18

**Authors:** Beatrice Dal Pino, Francesca Gorini, Melania Gaggini, Patrizia Landi, Alessandro Pingitore, Cristina Vassalle

**Affiliations:** 1 Fondazione Gabriele Monasterio CNR-Regione Toscana, 56124 Pisa, Italy; 2 Institute of Clinical Physiology, National Research Council, 56124 Pisa, Italy

**Keywords:** Lp(a), biomarkers, mortality, non-fatal myocardial infarction, coronary artery disease, prognosis, sex-related differences, residual risk, type 2 diabetes

## Abstract

Lipoprotein(a)-Lp(a), which retains proatherogenic and prothrombotic properties, may be modified by hormonal and metabolic factors. However, few studies have focused on differences related to sex and cardiometabolic risk factors in the relationship between Lp(a) and cardiovascular disease, especially in terms of prognosis. This study aimed at evaluating the predictive value of Lp(a) (cut-off 30 mg/dL) for hard events (HEs: mortality and non-fatal myocardial infarction) according to sex and cardiometabolic risk factors in 2110 patients (1501 males, mean age: 68 ± 9 years) undergoing coronary angiography for known or suspected coronary artery disease. There were 211 events over a median follow-up period of 33 months. Lp(a) > 30 mg/dL did not confer a worse prognosis on the overall population. However, Kaplan–Meier subgroup analysis evidenced a worse prognosis in type 2 diabetes (T2D) females with elevated Lp(a) (log-rank test: *p* = 0.03) vs. T2D males and no-T2D patients, but not in other high-risk cardiovascular states (e.g., smoking, hypertension, reduced left ventricular ejection fraction or obesity). After Cox multivariate adjustment, Lp(a) remained an independent determinant for HEs in the T2D female subgroup, conferring an HR of 2.9 (95% CI 1.1–7.7, *p* < 0.05). Lp(a) is therefore a strong independent predictor of HR in T2D women, but not in T2D men, or in noT2D patients.

## 1. Introduction

Lipoprotein(a) (Lp(a)) is a low-density lipoprotein containing a molecule of apolipoprotein(a) and apolipoprotein B-100, with a structure similar to LDL cholesterol as well as plasminogen [1].

This molecule is associated with the pathogenesis and development of atherosclerotic damage, in view of its conformation, which gives Lp(a) proatherogenic and prothrombotic properties [1]. Accordingly, results from epidemiological and genetic studies have suggested the role of high Lp(a) as a biomarker of residual atherosclerotic risk and cardiovascular disease (CVD) [2,3,4]. Subsequent studies reinforced the importance of Lp(a), which should be assessed in all patients with premature coronary artery disease (CAD) in the absence of major coronary risk factors [5]. 

The 2019 ESC/EAS guidelines on dyslipidemia indicate that Lp(a) should be measured at least once in every person’s lifetime, in order to identify individuals with very high inherited Lp(a) levels who are at very high risk for CVD [6]. In particular, the risk may significantly increase when Lp(a) is above 50 mg/dL [7], although other studies showed that values of Lp(a) >30 mg/dL are sufficient to increase the risk of cardiovascular events or all-cause mortality [8,9,10]. The function and atherogenicity of Lp(a) may be modulated by glycation, and is increased in diabetic patients [11,12]. Moreover, some studies suggest that estrogen may reduce Lp(a), which therefore increases in postmenopausal women and decreases in individuals on hormone replacement therapy (HRT) [13,14,15].

At present, few studies have explored sex differences in the relationship between serum Lp(a) and CVD, especially in terms of prognosis and secondary prevention, while the under-representation of women in most studies may indicate a gap in the evidence [16,17].

Therefore, the aim of this study was to assess the association between Lp(a) concentration and hard events (HEs: mortality and non-fatal myocardial infarction) in a large population of patients undergoing coronary angiography for known or suspected coronary artery disease (CAD) and in subgroups in relation to sex and in the presence of cardiometabolic risk factors, in particular type 2 diabetes (T2D).

## 2. Materials and Methods

### 2.1. Study and Population Characteristics

We conducted a longitudinal retrospective clinical cohort study to evaluate sex-related differences in cardiometabolic risk factors and survival in patients older than 50 years and admitted to the Cardiology Department of the Institute of Clinical Physiology, National Research Council, in Pisa, (Italy), who underwent coronary angiography for known or suspected CAD and were followed up for 10 years (2110 patients, 1501 males, mean age: 68 ± 9 years). All data were acquired in the setting of institutional assistance within clinical care purposes in a retrospective manner from our institution’s patient dataset (image database), containing clinical characteristics, previous history, CAD risk factors, comorbidities, laboratory and instrumental results, pharmacological therapies and post-discharge follow-up outcomes, and analyzed anonymously as an aggregated group, not individually [18]. Exclusion criteria were applied as follows: unavailability of Lp(a) results, severe systemic diseases including neoplasia, acute or chronic inflammatory disease, immunological disease, HRT (for women) and patient refusal or inability to supply written informed consent.

Data on smoking (no smokers, smoking history), arterial hypertension (systolic blood pressure > 140 mmHg and/or diastolic pressure > 90 mmHg or use of antihypertensive agents), T2D (fasting plasma glucose > 126 mg/dL or use of antidiabetic treatment), obesity (defined as body mass index > 30 kg/m^2^) and dyslipidemia (total cholesterol ≥ 200 mg/dL, triglyceride ≥ 150 mg/dL or current use of lipid-lowering therapy) were coded in a dichotomized manner. Previous medical therapy included angiotensin-converting-enzyme inhibitors, beta-blockers, lipid-lowering agents, antidiabetic agents, diuretics and aspirin.

The primary outcome was the occurrence of HEs. Follow-up was assessed by phone calls, personal communication with the patient’s physician or outpatient follow-up visits. Patients were followed from admission until the end point (mortality, the information on which was obtained from medical records or death certificates) or for a maximum of 10 years from the time of enrollment. The definition of cardiac death required the following documentation: significant arrhythmias, cardiac arrest, death attributable to congestive heart failure or myocardial infarction in the absence of any other precipitating factors. The diagnosis of myocardial infarction was based on documentation of persistent electrocardiographic ST segment changes, or the development of new Q waves, in association with elevation of laboratory biomarkers.

### 2.2. Statistical Analysis

Data are presented as number and percentages for categorical variables and as means and standard deviations or median where appropriate for continuous variables. Statistical analysis included χ^2^ tests for categorical variables. Estimates of survival probabilities were calculated using the Kaplan–Meier method and compared with the log-rank test. Variables were included in the multivariate Cox model based on significance in the univariate analyses to evaluate the effect of variables on survival time, reporting the hazard ratio (HR) with a 95% confidence interval of probability (95% CI). *p* values were two-sided with a significance threshold of 0.05. All statistical analyses were performed using Statview statistical package, version 5.0.1 (SAS Institute, Abacus Concept, Inc., Berkeley, CA, USA).

## 3. Results

Table 1 summarizes the study sample characteristics stratified by sex. Male patients were more likely to have a higher incidence of smoking history and were characterized by a higher proportion of subjects with reduced left ventricular ejection fraction (LVEF) (<50%), while female patients were likely to have a higher incidence of hypertension, CAD familiarity and obesity.

**Figure 1:**
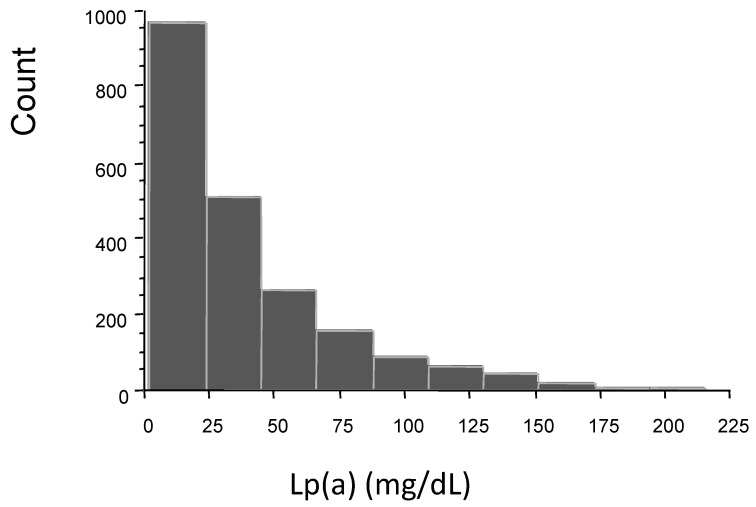
Lp(a) distribution in the overall population.

The distribution of Lp(a) in the cohort of patients was skewed to the right (Figure 1). The median level of lipoprotein was 27 mg/dL (29 and 26 mg/dL in female and male patients, respectively). 

In both sexes, the percentage of subjects with increased Lp(a) (cut-off of 30 mg/dL) differed depending on the presence of dyslipidemia (40 vs. 51%, *p* < 0.05 and 35 vs. 47%, *p* < 0.001 in females and males, respectively) and family history of CAD only in males (41 vs. 49%, *p* < 0.01) (Table 2).

**Figure 2:**
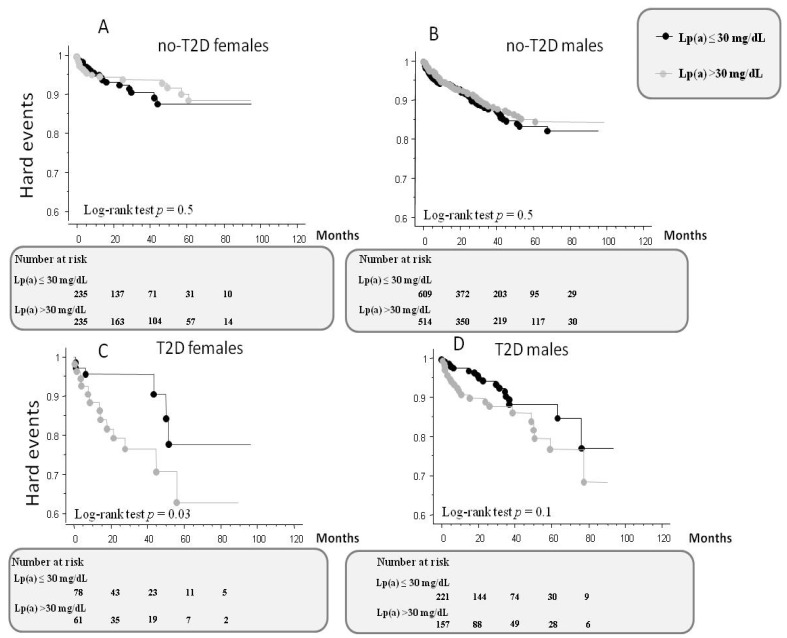
Kaplan–Meier survival curves according to Lp(a) levels in female and male patients with and without T2D (**A**–**D**), with hard events (mortality and non-fatal myocardial infarction) as end points.

Overall, 211 HEs (161 deaths and 50 non-fatal myocardial infarctions) were recorded during a mean follow-up of 33 months. As determined by Kaplan–Meier analysis, elevated Lp(a) levels (>30 mg/dL) did not lead to a worse long-term prognosis (log-rank test *p* value 0.68). However, subgroups analysis showed a worse prognosis in T2D females with elevated Lp(a) (log-rank test *p* value 0.03) (Figure 2C) compared to T2D/no-T2D males and no-T2D female patients (Figure 2A,B,D).

In female patients with T2D, Cox regression analyses revealed a significant association of Lp(a) with outcomes; in particular, high Lp(a) levels (>30 mg/dL) were associated with HEs with an HR of 2.9 (95% CI 1.1–7.7, *p* < 0.05) after multivariate adjustment (Table 3).

In our cohort, high-sensitivity C-reactive protein (hsCRP), fibrinogen and the erythrocyte sedimentation rate (ESR) were available in a subset of patients, showing the following correlations with logLp(a) values: logLp(a) vs. logCRP (correlation not significant in 588 female patients, r = 0.11 *p* < 0.001 in 1451 males); logLp(a) vs. logESR (r = 0.16, *p* < 0.001 in 595 female patients, r = 0.18 *p* < 0.001 in 1465 males); logLp(a) vs. logfibrinogen (correlation not significant in 571 female patients, r = 0.13, *p* < 0.001 in 1410 males). 

## 4. Discussion

The main result of this study is the independent association of Lp(a) levels with HEs in a cohort of T2D women undergoing coronary angiography for known or suspected CAD. Conversely, no relationship between events and Lp(a) levels in diabetic men or in non-diabetic subjects belonging to both sexes was observed.

Genetically predicted and measured Lp(a) values reveal a strong and consistent relationship with CAD risk and outcomes, identifying Lp(a) as an important element of residual cardiovascular risk [4,19]. In our overall population, Lp(a) > 30 mg/dL did not confer a worse prognosis, in contrast to several previous findings that identified Lp(a) as a prognostic independent risk factor for cardiovascular events, although it is important to remember that there is marked heterogeneity across studies evaluating the prognostic significance of Lp(a) [20]. This discrepancy may be due to different reasons. Importantly, the relationship between Lp(a) and CAD may vary depending on the patient’s baseline risk based on demographic characteristics (e.g., age, sex) and the presence/absence of coexisting CAD risk factors, such as inflammation and/or a procoagulant status [21,22,23,24]. 

Specifically, in the Multi-Ethnic Study of Atherosclerosis (MESA) Apolipoprotein ancillary dataset, isolated Lp(a) elevation was not associated with increased CVD risk, whereas the combination of elevated Lp(a) (≥50 mg/dL) and hsCRP (≥2 mg/L) was independently associated with CVD risk (HR, 1.62; 95% CI, 1.25–2.10) and all-cause mortality (HR, 1.39; 95% CI, 1.12–1.72) [21,22]. Conversely, the relationship between Lp(a) and cardiovascular events may be attenuated in patients with lower low-density lipoprotein cholesterol values [20]. The data we obtained in a subgroup of patients evidenced correlations between logLp(a) values and CRP, fibrinogen and the erythrocyte sedimentation rate (ESR), confirming the relationship between Lp(a) and these pathways, which was especially evident in male patients. Thus, it is important to evaluate the independent and combined association of Lp(a) and hsCRP or other biomarkers with cardiovascular outcome in specific population subgroups. Moreover, using different methods to quantify Lp(a) may influence the final results [25,26,27]. 

The choice of cut-off may also be a key factor, because when Lp(a) is evaluated as a categorical variable, the thresholds for the categories may differ between studies. However, we observed that Lp(a) was not significantly associated with HEs as assessed considering the per unit increase in LogLp(a) (HR of 1.28, 95% CI: 1–1.8, *p* = 0.16), and using the categorical threshold of 50 mg/dL (HR 1.05, 95% CI: 0.8–1.4, *p* = 0.7) in the univariate analysis in the overall population. 

In the context of T2D, Lp(a) is associated with T2D risk and CAD disease severity, as well as with microvascular and kidney complications and adverse events in patients with diabetes and elevated Lp(a) [28]. Lp(a) may increase the risk of the onset and development of T2D and the cardiometabolic burden via pro-atherogenic and pro-inflammatory effects, and induce a prothrombotic status through a variety of mechanisms (e.g., inhibition of the fibrinolytic system and enhancement of tissue-factor-mediated pathways). Conversely, a hyperglycemic status may promote the glycation of lipoproteins, while reduced insulin production can further exacerbate the increase in Lp(a) in T2D, as insulin inhibits hepatic apo(a) and apoB production through suppressed transcription [28].

Although sex differences in Lp(a)-related risk are still unclear, there is evidence that estrogen reduces Lp(a), as well as that Lp(a) rises in postmenopausal women, but its levels are reduced by HRT [29,30]. Accordingly, some data indicated that the relationship between elevated Lp(a) levels and increased CVD risk may be modulated by HRT, as the CV predictive role of Lp(a) in women not taking HRT was instead markedly attenuated in those taking HRT [31]. Women in the Nurses’ Health Study with Lp(a) levels > 30 mg/dL had an increased risk of CAD events, which appeared to be modulated by thrombosis and inflammation [32]. Always in the Nurses’ Health Study, T2D women with increased Lp(a) levels had a higher risk of developing CAD [33]. Previous findings also suggest that Lp(a) has a stronger association with coronary artery calcification in T2D females with respect to males with and without T2D or no-T2D women [30].

Taken together, these data may indicate that Lp(a) deserves to be evaluated as a potential risk predictor in high-risk T2D women. This finding may be of particular interest, as a woman’s CV risk still has unknown specific characteristics and remains largely underestimated. Thus, research on and identification of reliable additive biomarkers for optimized CV assessment and improvement in CV management in women are expected and welcome. Moreover, conventional therapeutic strategies (such as statins) have resulted in a substantially ineffective reduction in Lp(a), and in some cases may even increase its concentration [34]. Other drugs that lower Lp(a) levels (e.g., niacin or cholesteryl ester transfer protein inhibitors) do not show significant beneficial effects on cardiovascular outcomes. However, some tools currently reduce Lp(a) while also lowering CV risk (e.g., PCSK9 inhibitors and lipoprotein apheresis). For PCSK9 inhibitors, the magnitude of clinical benefit is related to baseline Lp(a) value and associated with the degree of Lp(a) decrease. Therefore, the identification of patients that may benefit most from such therapies as well as the extent of Lp(a) reduction required to benefit the CV system represents a very important challenge in targeting this biomarker as a component of residual cardiovascular risk. 

Other treatment options are currently available (e.g., gene silencing via RNA interference with use of antisense oligonucleotides or small interfering RNA molecules) that appear to reduce Lp(a) levels by more than 70%, and could be further evaluated for their reliability in reducing overall CV events in female patients with high-risk T2D [35]. 

### Strengths and Limitations

The study may present limitations related to its retrospective nature, and the single-center experience, although the number of patients enrolled is large (even for the female counterpart), and the focus on sex differences is an aspect often neglected in research studies and clinical practice. The sample size is not necessarily balanced between sexes, as it is well known that CAD is more common in men than in women (men usually have a 2-fold higher incidence of CAD and related mortality than women) [36]. A further limitation is the lack of availability of information on menopausal status in female patients, although we included patients over 50 (only 28 women between 50 and 55 years), which is the average age at which menopause occurs among women from industrialized countries; thus, the large majority of female patients in our cohort were postmenopausal [37]. However, these data highlight that there may be potential sex-related mechanisms underlying the relationship between Lp(a), T2D and CAD, since Lp(a) would appear to predict additive risk in the case of women with T2D. 

## 5. Conclusions

The concentration of Lp(a) is mainly determined by genetics (>90%), more than any other lipoprotein. In patients with suspected or known CAD, Lp(a) might represent an additive significant risk predictor in high-risk T2D women, but not in male patients with or without T2D or in non-diabetic women. Further studies are warranted to confirm our preliminary results and thus contribute to a better assessment of the risk profile of this specific population.

## Figures and Tables

**Table 1:** Characteristics of the studied population.

Variable	Females	Males	*p* Value
	*n* = 609	*n* = 1501	
Age (50th percentile: 70 years for females, 67 years for males)	330 (54)	769 (51)	ns
Type 2 Diabetes	139 (23)	378 (25)	ns
Hypertension	379 (62)	850 (57)	<0.05
Dyslipidemia	465 (76)	1180 (79)	ns
Familiarity with CAD	317 (52)	677 (45)	<0.01
Smoking History	143 (23)	750 (50)	<0.001
Obesity (<30 kg/m^2^)	172 (28)	323 (21)	<0.001
LVEF (<50%)	130 (21)	455 (30)	<0.001
Lp(a) (>30 mg/dL)	296 (49)	671 (45)	ns

Data are reported as number (%) in the female and male subgroups. Abbreviations: CAD: coronary artery disease; Lp(a): lipoprotein(a); LVEF: left ventricular ejection fraction; ns, not statistically significant.

**Table 2:** Number and percentage of subjects with Lp(a) > 30 mg/dL in female and male patients for each variable subgroup.

	Females		Males	
	*n* = 609		*n* = 1501	
Variable	Number of Subjects with Lp(a) > 30 mg/dL (%)	*p* Value	Number of Subjects with Lp(a) > 30 mg/dL (%)	*p* Value
Age (<50th percentile)	143 (51)	ns	337 (46)	ns
Age (≥50th percentile)	153 (46)		334 (43)	
no-T2D	235 (50)	ns	514 (46)	ns
T2D	61 (44)		157 (42)	
no-Hypertension	118 (51)	ns	294 (45)	ns
Hypertension	178 (47)		377 (44)	
no-Dyslipidemia	58 (40)	<0.05	111 (35)	<0.001
Dyslipidemia	238 (51)		560 (47)	
no-CAD Familiarity	138 (47)	ns	340 (41)	<0.01
CAD Familiarity	158 (50)		331 (49)	
no-Smoking History	222 (48)	ns	330 (44)	ns
Smoking History	74 (52)		341 (45)	
no-Obesity	210 (48)	ns	524 (45)	ns
Obesity	86 (50)		147 (45)	
LVEF (<50%)	67 (51)	ns	191 (42)	ns
LVEF (≥50%)	229 (48)		480 (46)	

Data are reported as the ratio between the number of individuals with Lp(a) > 30 mg/dL and the total number of subjects in each subgroup (%). Abbreviations: CAD: coronary artery disease; Lp(a): lipoprotein(a); LVEF: left ventricular ejection fraction; T2D: type 2 diabetes; ns, not statistically significant.

**Table 3:** Univariate and multivariate COX analysis for HEs in T2D female patients.

Predictors	Univariate Analysis		Multivariate Analysis	
	HR (95%CI)	*p* Value	HR (95%CI)	*p* Value
Age (50th percentile)	2.5 (1–7.1)	<0.05	2.7 (1–7.7)	0.05
Hypertension	1.4 (0.5–3.8)	ns	-	-
Dyslipidemia	1.8 (0.4–8.0)	ns	-	-
Familiarity with CAD	0.5 (0.2–1.2)	ns	.	-
Smoking History	1.2 (0.4–3.9)	ns	-	-
Obesity (<30 kg/m^2^)	0.7 (0.3–1.7)	ns	-	-
Left Ventricular Ejection Fraction (<50%)	1.6 (0.7–4.3)	ns	-	-
Lp(a) (>30 mg/dL)	2.7 (1–7.2)	<0.05	2.9 (1.1–7.7)	<0.05

Abbreviations: HR: hazard ratio; 95%CI: 95% confidence interval of probability; ns, not statistically significant.

## Data Availability

Data may be provided by the authors upon (reasonable) request.

## Abbreviation

Lp(a)	lipoprotein(a)
CAD	coronary artery disease
T2D	type 2 diabetes
CVD	cardiovascular disease
HEs	hard events
HRT	hormone replacement therapy
LVEF	left ventricular ejection fraction
hsCRP	high-sensitivity C-reactive protein
ESR	erythrocyte sedimentation rate
HR	hazard ratio
95% CI	95% confidence interval of probability
